# Acetyl-CoA synthetase mutations affect the susceptibility of *Plasmodium falciparum* to antimalarial drugs

**DOI:** 10.1128/spectrum.01026-25

**Published:** 2025-09-11

**Authors:** Wei Zhao, Zheng Xiang, Weilin Zeng, Yucheng Qin, Maohua Pan, Yanrui Wu, Mengxi Duan, Ye Mou, Tao Liang, Yanmei Zhang, Cheng Liu, Xiuya Tang, Yaming Huang, Gongchao Yang, Liwang Cui, Zhaoqing Yang

**Affiliations:** 1 Yunnan Provincial Key Laboratory of Public Health and Biosafety & Department of Pathogen Biology and Immunology, Faculty of Basic Medical Science, Kunming Medical University, Kunming, Yunnan, China; 2 Department of Clinical Laboratory, the Affiliated Hospital of Yunnan University, Kunming, Yunnan, China; 3 Department of Infectious Diseases, Shanglin County People’s Hospital, Chinese Center for Tropical Diseases Research Ruijin Hospital Affiliated to Shanghai Jiao Tong University School of Medicine Clinical Research Alliance for Parasitic Diseases Related Infectious Diseases, Nanning, Guangxi, China; 4 Department of Infectious Diseases and Hepatology, The First Affiliated Hospital of Kunming Medical University, Kunming, Yunnan, China; 5 Department of Protozoan Diseases, Guangxi Zhuang Autonomous Region Center for Disease Prevention and Control, Nanning, China; 6 Department of Advanced Biomedical Education, University of Mississippi Medical Center, Jackson, Mississippi, USA; 7 Department of Internal Medicine, Morsani College of Medicine, University of South Florida 7831, Tampa, Florida, USA; University of Illinois Urbana-Champaign, Urbana, Illinois, USA

**Keywords:** Acetyl-CoA synthetase, *Plasmodium falciparum*, artemisinin, drug resistance

## Abstract

*Plasmodium falciparum* acetyl-CoA synthetase (PfAcAS) is an important source of acetyl-CoA. We detected mutations S868G and V950I in PfAcAS by whole-genome sequencing analysis in certain recrudescent parasites after treatment with artesunate and dihydroartemisinin-piperaquine. Using CRISPR/Cas9 technology, we engineered parasite lines to carry the PfAcAS S868G and V950I mutations in two genetic backgrounds and evaluated their susceptibilities to antimalarial drugs *in vitro*. The results demonstrated that PfAcAS S868G and V950I mutations alone or in combination affected the susceptibility of *P. falciparum* to several antimalarial drugs, including the artemisinin derivatives (dihydroartemisinin, artesunate, and artemether) and chloroquine, although absolute changes in susceptibilities were modest.

**IMPORTANCE:**

Malaria, an infectious disease caused by *Plasmodium* parasites and transmitted by mosquitoes, continues to be one of the most pressing public health challenges worldwide. *P. falciparum* has demonstrated reduced sensitivity to artemisinin-based combination therapies (ACTs), thereby intensifying the difficulties associated with malaria management. Currently, only a limited number of molecular markers exist for identifying drug resistance in *P. falciparum*, and these markers do not fully elucidate the mechanisms behind this resistance. In this study, we performed whole-genome sequencing analysis on *P. falciparum* strains that reemerged following ACT treatment. We aim to identify molecules potentially associated with drug resistance, which may provide new molecular markers for monitoring drug resistance in *P. falciparum*.

## INTRODUCTION

Malaria, an infectious disease caused by *Plasmodium* parasites and transmitted by mosquitoes, remains one of the most significant global public health problems. *Plasmodium falciparum* has shown reduced sensitivity to artemisinin-based combination therapies (ACTs) in Southeast Asia (1–8) and Africa (9–15), making the management of malaria challenging. Artemisinin partial resistance (ART-R) in *P. falciparum* is manifested as delayed parasite clearance. After ACT treatment, sensitive parasites are cleared within 2 days, whereas infections associated with ART-R can persist for three or more days (16). *In vitro*, ART-R in *P. falciparum* is measured by the ring-stage survival assay (RSA) of 0–3-h ring-stage parasites after exposure to 700 nM dihydroartemisinin (DHA) for 6 h (17).

Mutations in the propeller domain of the PfK13 gene are major determinants of ART-R (18, 19). However, this marker for ART-R does not fully explain its complexities. There is evidence that parasites may exhibit ART-R without PfK13 mutations, suggesting the involvement of additional factors (20, 21). Therefore, it is urgent to explore additional molecular markers associated with ART-R.

*P. falciparum* isolates associated with recurrent parasitemia after ACT treatment were collected in our previous study from Chinese migrant workers who returned from Central and West Africa. These patients experienced treatment failures, as evidenced by recurrent parasitemia within 42 days post-treatment, despite having received intravenous artesunate and orally administered DHA-piperaquine (PPQ) (Table S1) (22). Here, we used genomics to explore potential genetic changes associated with the recrudescent parasites. We identified two point mutations in the *P. falciparum acetyl-CoA synthetase* (*PfAcAS*) gene with significantly increased prevalence in the recrudescent parasites. Engineering these mutations in two different *P. falciparum* strains demonstrated that they could confer reduced *in vitro* susceptibilities to several commonly used antimalarial drugs, including the ART derivatives, although absolute changes in susceptibilities were modest.

## MATERIALS AND METHODS

### Study design

Blood samples were collected from 24 patients infected with *P. falciparum* in Shanglin County, Guangxi, between 2016 and 2018. These included 11 patients with recurrent parasitemia (case group) and 13 patients who showed adequate clinical and parasitological responses (ACPRs) to treatment (control group). All patients developed malaria-like symptoms within 1–2 weeks after returning to China and were diagnosed by microscopic examination of Giemsa-stained thick and thin blood smears. At the time of evaluation, there was no local transmission of malaria (22). Patients with uncomplicated *P. falciparum* malaria were invited to donate 2–3 mL of venous blood. Blood samples were collected in tubes containing sodium citrate as an anticoagulant and transported at 4°C to the laboratory for culture adaptation.

All 24 patients initially received intravenous (IV) artesunate injections. After clinical improvement, they were switched to oral artemisinin-based combination therapy (ACT), specifically DHA-PPQ (DHA 40 mg–PPQ 320 mg), for further treatment. Briefly, patients received either 3 or 7 days of IV artesunate (120 mg per day or 2.4 mg/kg), followed by a 3-day course of DHA–PPQ (Table S1) (22). Among them, 11 patients experienced treatment failure, with recurrent parasitemia detected within 42 days post-treatment, despite receiving both IV artesunate and oral DHA–PPQ. Blood samples from these patients were collected before treatment initiation upon recrudescence and assigned to the case group. The remaining 13 strains that showed ACPRs were assigned to the control group (Table S1). Strains from the MalariaGen Pf7 data set (https://www.malariagen.net/parasite/) included an additional 110 ART-sensitive strains that were also utilized as controls (Table S2).

### SNP identification and quality filtering

The genomic DNA of parasites was extracted using the QIAamp DNA Blood Mini Kit (Qiagen). All libraries sequenced on the Illumina NovaSeq 6000 were subjected to paired-end reads of 150 bp. High-quality short reads were mapped on the 3D7 reference genome from the PlasmoDB database (23). All sequenced raw reads were filtered by removing the adapter and low-quality sequences with Trimmomatic-3.0 (24) and mapped to the 3D7 reference sequence using Burrows-Wheeler Aligner (25). Genotyping was performed using an in-house R script based on GATK4 best-practice workflows and recalibrated with pf3k standard known-site files (26). High-quality SNPs were derived by excluding SNPs with >5% missing calls in each sample; missing calls were defined as positions with <2 reads. Additionally, we downloaded 110 *P*. *falciparum* whole-genome sequences from MalariaGen Pf7 data set (https://www.malariagen.net/parasite/) and added them to the control group. These sequences originated from western African *P. falciparum* isolates collected in 2013 and 2014, including Ghana (*N* = 70), Congo (*N* = 30), and Cameroon (*N* = 10). The geographical distribution of these samples was considered, taking 10 times the sample size of the recrudescent parasites (Table S1). For the genome-wide association study (GWAS), we applied PLINK v1.9 with the following parameters: --maf 0.01 --hwe 0.05 --mind 0.1 --assoc, we identified non-synonymous mutation sites with a *P*-value of 2.5 × 10^−11^, which is less than the significance threshold defined by Bonferroni correction (*P* ≤ 7.63 × 10^−7^).

### Plasmid construction and preparation

**Fig 1:**
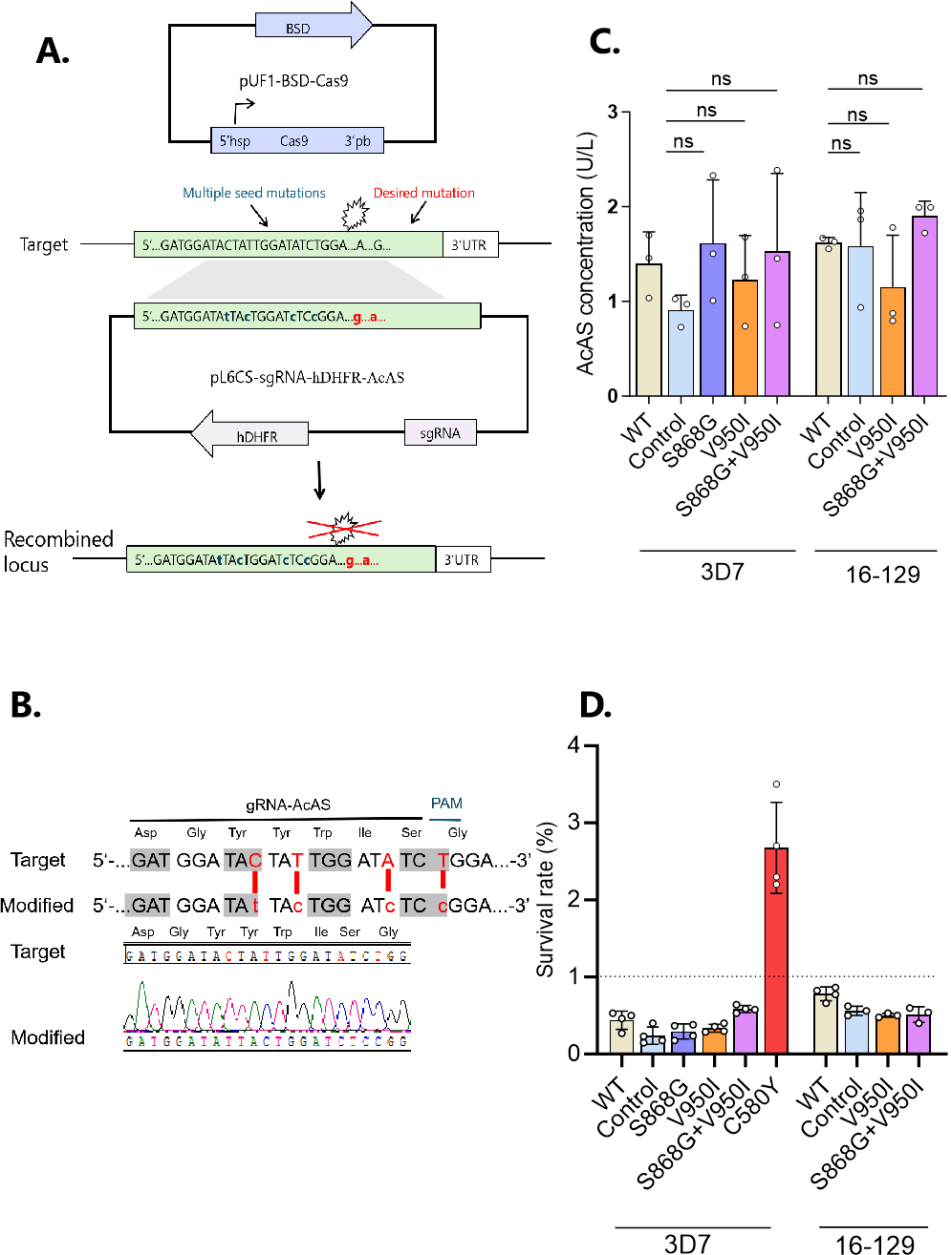
(**A**) A schematic diagram illustrates gene editing of PfAcAS using CRISPR/Cas9. Donor fragment of 1.03 kb was designed with the desired point mutation in the center (shown in red) and additional silent mutations (shown in blue) in the gRNA target sites marked by a lightning bolt. These additional mutations are important to prevent cleavage of the modified locus as well as the plasmid. The donor and the gRNA are cloned into the all-in-one plasmid pL6CS-gRNA-hDHFR, while pUF1-BSD-Cas9 is designed to express the Cas9 protein, including selection mark BSD. (**B**) The 20 bp gRNA and PAM (TGG) in the target sequence. The modified sequence contains synonymous mutations as shield mutations to prevent repeated cutting in the transgenic strains. (**C**) AcAS concentration of wild-type and mutant parasites in the 3D7 and 16-129 backgrounds. Statistical comparisons were made between edited parasite lines with their respective control using a one-way ANOVA test (*P* > 0.05). Three biological replicates were performed for each parasite isolate. (**D**) Ring-stage survival rates of wild-type and edited parasites in the 3D7 and 16-129 backgrounds. The dotted line indicates the 1% threshold used to define artemisinin partial resistance.

The construction of the pL6CS-sgRNA-hDHFR-AcAS and pUF1-BSD-Cas9 plasmids was performed following the previous report (27). Based on pL6CS-sgRNA-hDHFR, we constructed pL6CS-sgRNA-hDHFR-AcAS-Control/S868G/V950I/S868G + V950I plasmids. The plasmids harbored the sgRNA and donor DNA sequence; the latter carried sgRNA shield mutations and the desired mutation (Fig. 1A). Briefly, pL6CS-sgRNA-hDHFR was initially digested by *Xhol I* and *Avr II* (NEB, USA) restriction enzymes. The sgRNA primers (Table S6) were synthesized to form double-stranded DNA. The resulting product was diluted 20-fold and subjected to recombination with the plasmid mentioned above for 30 min at 37°C, following the instructions of ClonExpress II One Step Cloning Kit (C112) (Vazyme, China). The sgRNA was inserted into the plasmid. The donor carrying either S868G or V950I mutation was amplified using primers listed in Table S7. All primers contained the desired mutations or 20 bp necessary for homologous arms. High-fidelity polymerase (Vazyme, China) was used for all PCR amplifications, following the recommended protocols. The resulting fragments were then assembled to generate the donor fragments. Subsequently, the pL6CS-sgRNA-hDHFR plasmids and donor fragments underwent digestion with the *Acs I* and *Afl II* restriction enzymes. The resulting products were purified, and the linearized plasmids with different donor fragments were mixed separately in a recombinant enzyme system. The mixing was carried out for 30 min at 37°C. This process was performed to construct the pL6CS-sgRNA-hDHFR-Control/S868G/V950I/S868G + V950I plasmids. Then, the pL6CS-sgRNA-hDHFR-AcAS plasmids were transformed into XL-10 gold-competent cells (Weidibio, China). Plasmids were then extracted using the Plasmid Mini Kit (Omega, USA) and confirmed by sequencing.

### *P. falciparum* culture and transfection

Parasites 3D7 and 16-129 were cultured in type O erythrocytes and grown in a complete medium containing 10.4 g/L RPMI 1640, 2 g/L NaHCO_3_, 0.1 mM hypoxanthine, 50 mg/L gentamycin and 5 g/L Albumax II, 25 mM HEPES. The cultures were incubated at 37°C in a gas mixture of 5% CO_2_ and 5% O_2_ (28). The 16-129 strain, which was collected from a case showing ACPR after ACT treatment, served as the background strain. Similar to some recurrent parasitemia samples collected in this study, they all originated from the African region, were returned from Ghana in 2016, underwent clinical treatment with ACTs, and were subsequently adapted to culture in the laboratory. Transfection was performed in pre-loaded red blood cells (RBCs) (29, 30). Briefly, the parasites were synchronized using 5% sorbitol and cultured until the schizont stage. At this point, 60% Percoll was employed to isolate the schizonts for transfection. Then, fresh RBCs were washed with 1× Cytomix solution just before transfection. The transfection mixture contained 100 µg pUF1-BSD-Cas9 plasmids, 100 µg pL6CS-sgRNA-hDHFR-AcAS plasmids, and 160 µL of 100% washed RBCs, with the final volume adjusted to 400 µL with Cytomix. A Bio-Rad Gene Pulser was used for transfection, with the following settings: voltage (310 V), capacity (950 µF), electric resistance (infinite), and cuvette gap (2 mm). After the transfection, the broken RBCs were washed away from the mixture, and the remaining cells were transferred into flasks with fresh media. Parasite schizonts were added to invade the RBCs pre-loaded with plasmids. On the 4th day post-transfection, Blasticidin S HCl (BSD) (MCE, USA) and WR99210 (Thermo, USA) drugs were added to the medium at final concentrations of 2 µg/mL BSD and 2.5 nM WR99210, respectively. The medium was changed every other day until the parasites reappeared when they were confirmed by PCR and Sanger sequencing. Subsequently, parasite dilution cloning was performed, and parasites carrying the desired mutations were selected using drugs (WR99210 and BSD).

### Assessing AcAS concentration in *P. falciparum*

The AcAS enzyme-linked immunosorbent assay (ELISA) kit (MEIMIAN, China) was utilized to measure the protein levels of PfAcAS. After 30 min of equilibrium at room temperature, the stock was diluted in the standard diluent and added to a 96-well AcAS antibody-coated plate at the starting concentration of 60 U/L, which was serially diluted with a range of 0.93–60 U/L. Firstly, 50 µL of standard solutions at different concentrations was added to the coated plate. Then, 40 µL of sample diluent solution and 10 µL of sample proteins were added to the sample wells, resulting in a final sample dilution of five times. Each sample was tested in three replicates. After incubating at 37°C for 30 min, the plate was washed five times with the wash buffer. Next, an AcAS antibody labeled with horseradish peroxidase was added and incubated for 30 min. Subsequently, the chromogenic agent was added and incubated in the dark for 10 min. The reaction was then stopped by adding the termination solution, and the readings were taken at a wavelength of 450 nm using a plate reader. The AcAS protein levels were calculated based on the standard curve.

### Ring survival rates (RSA)

RSA was performed as described previously (17, 31). Briefly, tightly synchronized 0–3-h ring-stage parasites were prepared at 1% parasitemia and 2% hematocrit, treated with 700 nM of DHA or the same concentration of solvent (DMSO) for 6 h, followed by washing off the drug and culturing the parasites for additional 66 h. Thin blood smears were made to assess the ring survival rates of the strains by microscopy, with 10,000 RBCs counted on each slide. The ring survival rates were determined by comparing the number of surviving parasites in DHA-treated wells with those in vehicle-treated wells. Each parasite isolate was tested in three biological replicates.

### *In vitro* drug susceptibility assays

Ten antimalarial drugs, including DHA, artemether, artesunate, naphthoquine, mefloquine, lumefantrine, PPQ, pyronaridine, chloroquine (CQ), and quinine, were used in the *in vitro* drug susceptibility assays. The SYBR Green I assay was conducted following the previous reports (32, 33). Briefly, synchronized parasites were adjusted to 0.5% parasitemia and 2% hematocrit at 37℃ for 72 h in the presence of the respective drugs for the susceptibility assays. The half-maximal inhibitory concentration (IC_50_) was estimated using a non-linear regression model implemented in GraphPad Prism 6.0.

### Statistical analysis

We used the one-way analysis of variance (ANOVA) test to compare data between the two groups. A *P* value of less than 0.05 was considered statistically significant.

## RESULTS

### Whole genome sequencing (WGS) of recrudescent *P. falciparum*

To understand potential mechanisms leading to recrudescence of *P. falciparum* parasitemia after ACT treatment, we performed WGS for the 11 recrudescent isolates following DHA-PPQ treatment and 13 isolates with ACPR collected at the same time from patients returning to China from Central and West African countries (Cameroon, Congo, and Ghana) (Table S1).

Meanwhile, sequences of 110 *P*. *falciparum* isolates, also from these three countries, were downloaded. We performed genomic comparisons between the 11 isolates with recrudescence (the case group) and 13 ACPR isolates plus the 110 isolates from the online database (the control group). For the GWAS analysis, we applied PLINK v1.9 and used Manhattan plots to show the significance of SNP association in the GWAS (Fig. S1). Of the 65,460 single nucleotide polymorphisms (SNPs) identified, 503 significantly differed between the two groups (below the Bonferroni correction *P* value of 7.63 × 10^−7^). Among them, 101 are non-synonymous mutations (Table S3). These 101 mutations are distributed across 89 genes, of which 36 have unknown functions. Gene ontology (GO) analysis on the 53 genes with known functions showed enrichment in nuclear transport, chromatin organization, endomembrane system, apical part of the cell, ubiquitin protein ligase activity, and GTPase activity (Table S4).

One of the genes identified from the genomic comparison is the *acetyl-CoA synthetase* (*PfAcAS*) gene (*PF3D7_0627800*). In the 11 recrudescent samples (case group), PfAcAS mutations S868G (0.29) and V950I (0.25) were significantly more prevalent than in the control group (both mutations were at 0.008). Additionally, the prevalence of these two loci in the public database is 0.01 (Table S3). Notably, these two mutations did not appear in the 13 ACPR isolates collected from returning travelers. Mapping these two mutations to the homologous model derived from the *Cryptococcus neoformans* AcAS structure revealed that they are not located near the active site of the enzyme, in particular, the predicted CoA binding site (Fig. S2). We also predicted structural domains using the SMART database, which revealed that the PfAcAS protein contains several low-complexity regions and several conserved adenosine monophosphate (AMP) binding motifs. S868G is located within one AMP-binding motif (positions 825–882), while V950I is near the terminal domain (positions 899–948).

### Engineering of the mutations using CRISPR/Cas9

**Fig 2:**
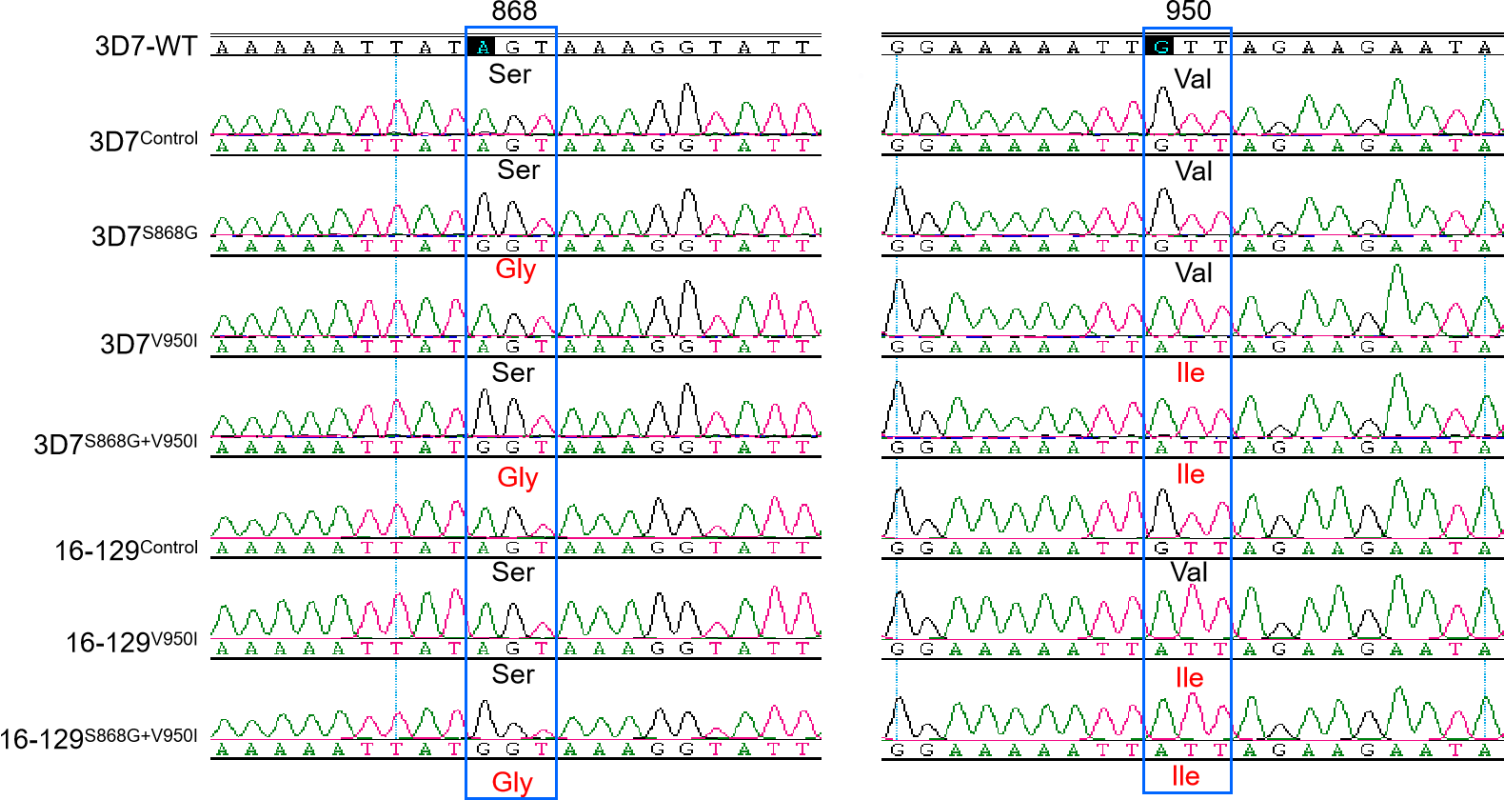
Sequencing of the PfAcAS fragments in edited parasites in the 3D7 and 16-129 genetic backgrounds. The positions of the amino acids are boxed, and the mutations are highlighted in red color.

PfAcAS is a viable antimalarial drug target, and *in vitro* evolution of resistance identified mutations in this gene to confer resistance to two drug-like compounds in the Medicines for Malaria Venture (MMV) library (26). This prompted us to investigate whether the two PfAcAS mutations identified in the recrudescent parasites could mediate drug resistance. We used the CRISPR/Cas9 technology to engineer the S868G and V950I variants in two genetic backgrounds (Fig. 1A and B): 3D7 and 16-129. The S868G and V950I mutations were introduced individually or together to create parasite clones 3D7^S868G^, 3D7^V950I^, 3D7^S868G+V950I^, 129^V950I^, and 129^S868G+V950I^ (Fig. 2). As controls, we also edited the 3D7 and 16-129 strains to carry synonymous changes at these two sites (3D7^Control^ and 129^Control^) (Fig. 2). Successful editing was verified by PCR of the *PfAcAS* fragments and sequencing, and the edited parasites were subsequently cloned.

To determine whether genetic modifications affected the expression of PfAcAS, we synchronized the parasite clones and assessed PfAcAS protein levels in the ring stage using an ELISA. The results indicated that there was no significant difference in the PfAcAS level between the mutant and the wild-type (WT) clones in both the 3D7 and 16-129 strains (*P*＞0.05) (Fig. 1C).

### *In vitro* susceptibility to antimalarial drugs

We next evaluated whether introducing the two mutations affected the parasite’s susceptibility to commonly used antimalarials. First, we performed RSA, including the 3D7^C580Y^ strain as a positive control for ART-R. Our results showed that while the 3D7^C580Y^ parasites had a survival rate of 2.68%, all engineered strains carrying the PfAcAS mutations had survival rates below 1% (0.24%–0.78%) (Fig. 1D; Table S5).

**Fig 3:**
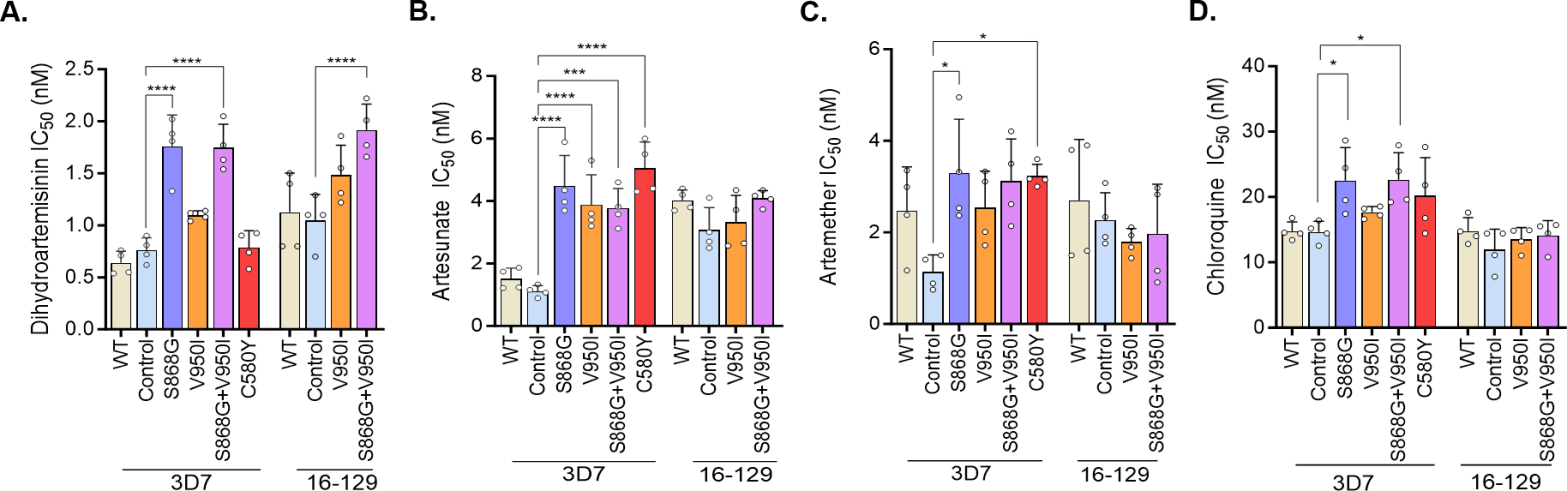
The IC_50_ values of the wild-type and mutant parasites against four antimalarial drugs, dihydroartemisinin (A), artesunate (B), artemether (C), and chloroquine (D). Statistical analysis was performed between mutant parasites and their respective controls (ANOVA). Four biological replicates were performed for each parasite isolate. *, ***, and **** represent *P* < 0.05, *P* < 0.001, and *P* < 0.0001, respectively.

Next, we evaluated the susceptibilities of edited parasite clones to 10 antimalarial drugs using the 72-h SYBR Green I assay. For DHA, the geometric mean IC_50_ values of S868G and S868 + V950I mutant strains were significantly higher than control strains in the 3D7 background (*P* < 0.0001) (Fig. 3A; Table S5). In the 16-129 background, only 129^S868G+V950I^ had a significantly higher IC_50_ value than the 16-129 control strain (*P* < 0.0001) (Fig. 3A; Table S5). Similarly, in 3D7, the artesunate IC_50_ values of all mutant strains were significantly higher than that of the control strain (*P* < 0.0001) (Fig. 3B; Table S5). However, such differences were not observed in the 16-129 background (Fig. 3B; Table S5). For artemether, only the 3D7^S868G^ had a much higher IC_50_ value than the 3D7^Control^ (*P* < 0.05) (Fig. 3C; Table S5), whereas there was no significant difference among the clones in the 16-129 background. Interestingly, two edited strains in the 3D7 background, 3D7^S868G^ and 3D7^S868G+V950I^, also showed significantly increased IC_50_ values to CQ, albeit these values were still below the reported cutoff value of 100 nM for resistance (Fig. 3D; Table S5). For the rest of the drugs tested, the edited parasites in both genetic backgrounds showed no significantly altered IC_50_ values compared with the control parasites (Fig. S3; Table S5).

## DISCUSSION

Through WGS, we have identified SNPs whose frequencies were significantly higher in recrudescent isolates after ACT treatment, suggesting potential roles in mediating ART-R. We were particularly interested in the S868G and V950I mutations in PfAcAS, as it is a viable drug target. PfAcAS is a critical enzyme necessary for the survival of parasites, and knockout or inhibition of the PfAcAS gene affects the growth of malaria parasites (34). Furthermore, mutations (A597V, T648M) in this gene were associated with resistance to drug candidates in the MMV drug development pipeline (35). However, in our study, we did not observe these two loci in the 11 recrudescent parasite strains. A recent study on MMV compounds evaluated four potential inhibitors targeting PfAcAS. Sequencing of *Plasmodium falciparum* isolates from Uganda revealed a high degree of polymorphism in the PfAcAS gene, identifying 43 different nonsynonymous SNPs. The prevalence of the S868G and V950I mutations of interest in Ugandan isolates also was observed, with S868G occurring at less than 1% and V950I approaching 3%. However, none of the observed polymorphisms were associated with decreased susceptibility to any of the studied PfAcAS inhibitors (36). We retrieved variation data for these two loci from the PlasmoDB database, which revealed that mutations at these sites were rare (< 0.01) (Table S3). In our study, we found that the prevalence of PfAcAS mutations S868G and V950I was significantly higher than that of the control group and the prevalence reported in public databases. We speculate that this result may be due to the small sample size in our study, and other loci identified also exhibited similar results. However, there is currently no evidence on how these mutations affect the susceptibility of malaria parasites to antimalarial drugs. Although the two mutations identified in this study are not located near the catalytic sites of the enzyme, the S868G mutation is located within one AMP-binding motif, potentially affecting the enzyme function. By engineering these mutations in 3D7 and a more recent clinical isolate from Ghana (16-129), we found that the S868G mutation was associated with reduced *in vitro* susceptibility of the edited parasites to artemisinin derivatives, and the effect is more notable in the 3D7 background.

The results of RSA with young ring-stage (0–3 h) parasites correlate with the *in vivo* ART-R phenotype—delayed parasite clearance (17). In our study, the parasite survival rates of the engineered parasite lines were all below 1%, the threshold used for ART-R, suggesting that the two PfAcAS mutations are not major drivers of recrudescence. This also highlights the need to characterize the additional mutations identified to have increased prevalence in the recrudescent isolates after ACT treatment (Table S3). Notably, many mutations are identified in genes with critical enzyme activities (Table S4).

We also assessed parasites’ susceptibilities to 10 commonly used antimalarial drugs using the 72-h standard drug assay. Interestingly, we found that the S868G mutation conferred significantly increased IC_50_ values to all three ART derivatives in the 3D7 background (not tested in the 16-129 background). We argue that the V950I mutation does not have such an effect because (i) this mutation alone did not affect parasites’ susceptibilities to all antimalarials tested in both genetic backgrounds (with a single exception to artesunate in 3D7) and (ii) the two mutations in combination did not alter the parasite’s susceptibilities to ARTs compared with the S868G mutation alone. In addition, it appeared that the S868G mutation might confer increased resistance to CQ, although the increase was relatively marginal. It is noteworthy that the *P. falciparum* CQ resistance transporter (*Pfcrt*) gene, the major determinant of CQ resistance, is wild type in both 3D7 and 16-129. These results indicate that the PfAcAS S868G and V950I mutations alone or in combination decreased the susceptibility of *P. falciparum* to several antimalarial drugs, including artemisinin derivatives and chloroquine, but that the absolute changes in drug susceptibility were modest, and that the mutations were not clearly linked to ART-R.

One caveat of this study is that the genomic comparison was based on only 11 parasite isolates with late parasitological failures (recrudescence), and responsible mutations may not be robustly identified. Nonetheless, the mutations identified in many critical cellular pathways serve as an important basis to probe their potential involvement in ART-R. Since the PfAcAS is among the 89 genes identified to have non-synonymous mutations, it is possible that ART-R may be a multi-gene phenomenon, with other significant mutations (Table S3) potentially working together to influence drug resistance. This study also shows that the PfAcAS mutations impose background-dependent effects on drug susceptibility, as has been found with PfK13 mutations (12, 37). Thus, future studies should evaluate additional mutations in diverse genetic backgrounds. Further investigation is required to confirm the significance of these candidate mutations.

## Data Availability

The datasets used in this study are available in NCBI and EBI, with the following accession numbers: ERR636260-ERR636269, ERR636083, ERR636091, ERR636098, ERR1214127, ERR1214129-ERR1214133, ERR1214135-ERR1214143, ERR1214147-ERR1214171, ERR1214174, ERR1214175, ERR1214177, ERR1214178, ERR1214183, ERR1214184, ERR1214186, ERR1214187, ERR1214189-ERR1214191, ERR1214195, ERR1214197, ERR1214198, ERR1214200, ERR1214201, ERR1214206, ERR1514577-ERR1514583, ERR1514588-ERR1514591, ERR1514594, ERR1514596-ERR1514605, ERR1514607-ERR1514610, ERR1514612-ERR1514615, ERR580582, ERR580472, ERR562829, ERR580473, ERR580509, ERR580542, ERR580580, ERR580544, ERR580545, ERR580471, SRR33420179-SRR334202.

## SUPPLEMENTAL MATERIAL

Fig. S1 spectrum.01026-25-s0001.tif Manhattan plots showing the significance of SNP association in the GWAS.

Fig. S2 spectrum.01026-25-s0002.tif Homology modeling of PfAcAS reveals that mutations identified are not clustered around the active site of the enzyme or the CoA binding site.

Fig. S3 spectrum.01026-25-s0003.tif IC50 values of wild-type and mutant parasites for six antimalarial drugs.

Supplemental material spectrum.01026-25-s0004.docx Supplemental figure legends.

Supplemental tables spectrum.01026-25-s0005.docx Tables S1 to S7.

ASM does not own the copyrights to Supplemental Material that may be linked to, or accessed through, an article. The authors have granted ASM a non-exclusive, world-wide license to publish the Supplemental Material files. Please contact the corresponding author directly for reuse.
