# Supplementary figures and images for: Acetyl-CoA synthetase mutations affect the susceptibility of Plasmodium falciparum to antimalarial drugs
Source: Microbiol Spectr. 2025 Sep 11;13(10):e01026-25. doi: 10.1128/spectrum.01026-25 (PMC12502573; doi:10.1128/spectrum.01026-25)

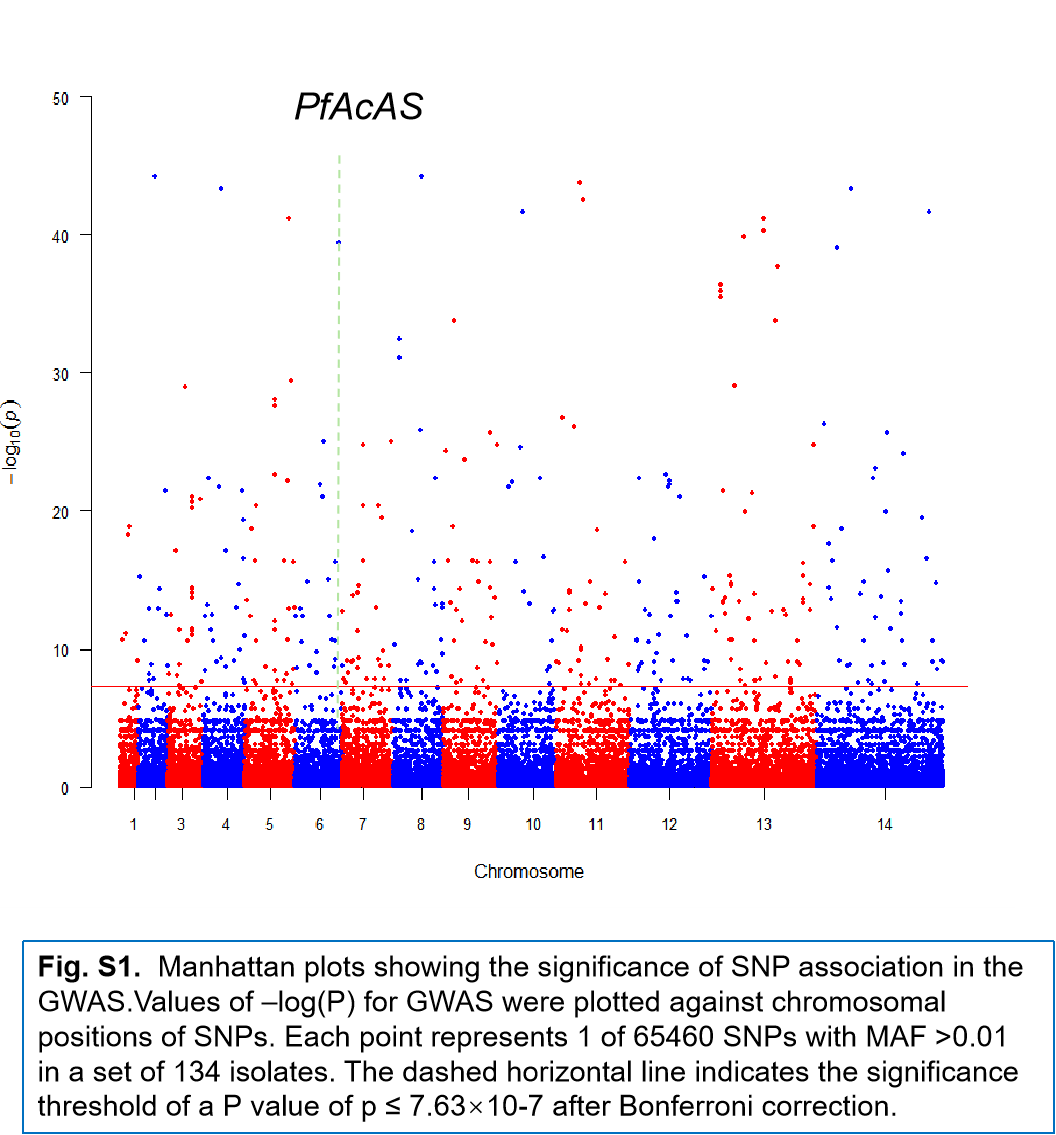

Supplement: Fig. S1 — Manhattan plots showing the significance of SNP association in the GWAS. [file spectrum.01026-25-s0001.tif]

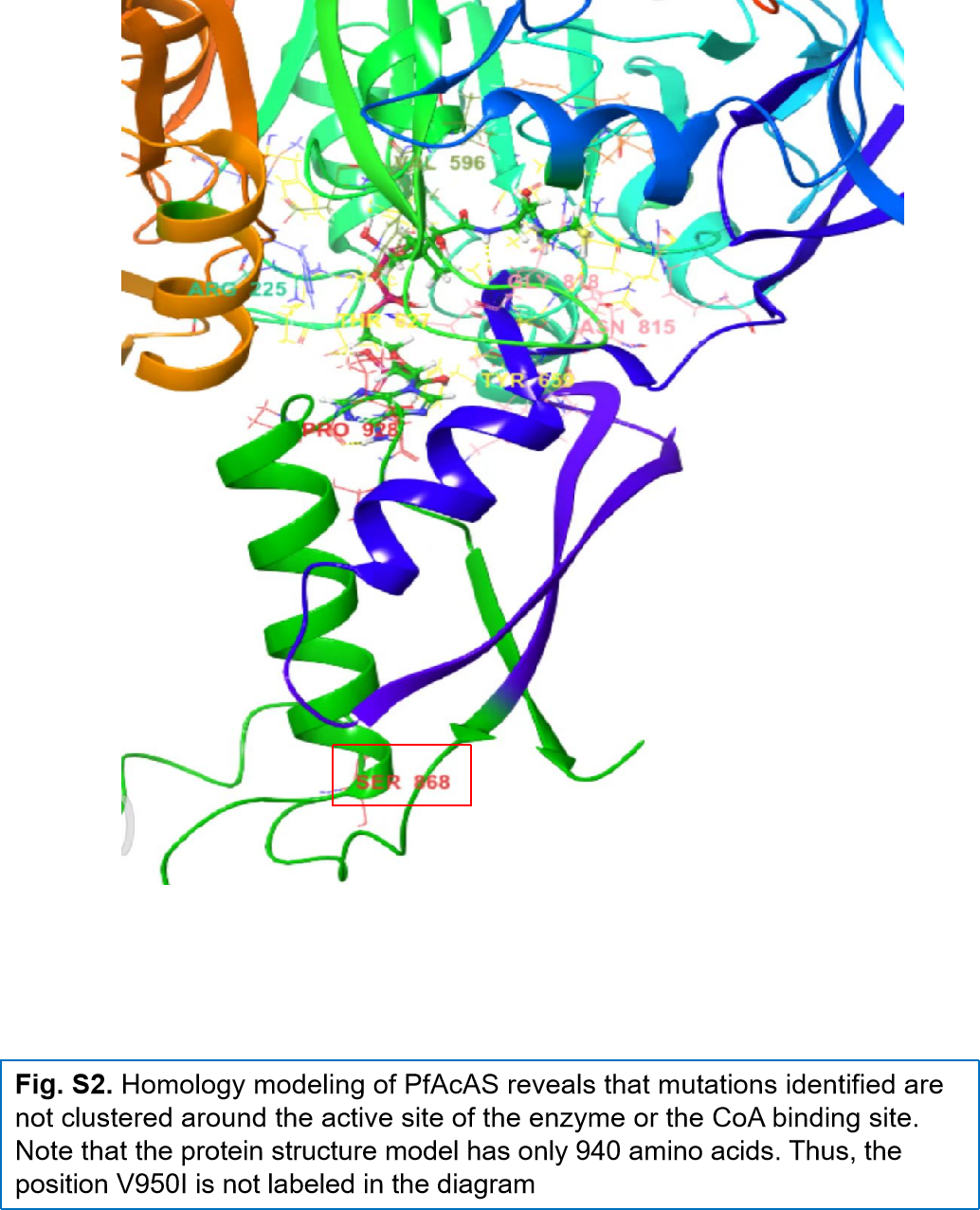

Supplement: Fig. S2 — Homology modeling of PfAcAS reveals that mutations identified are not clustered around the active site of the enzyme or the CoA binding site. [file spectrum.01026-25-s0002.tif]

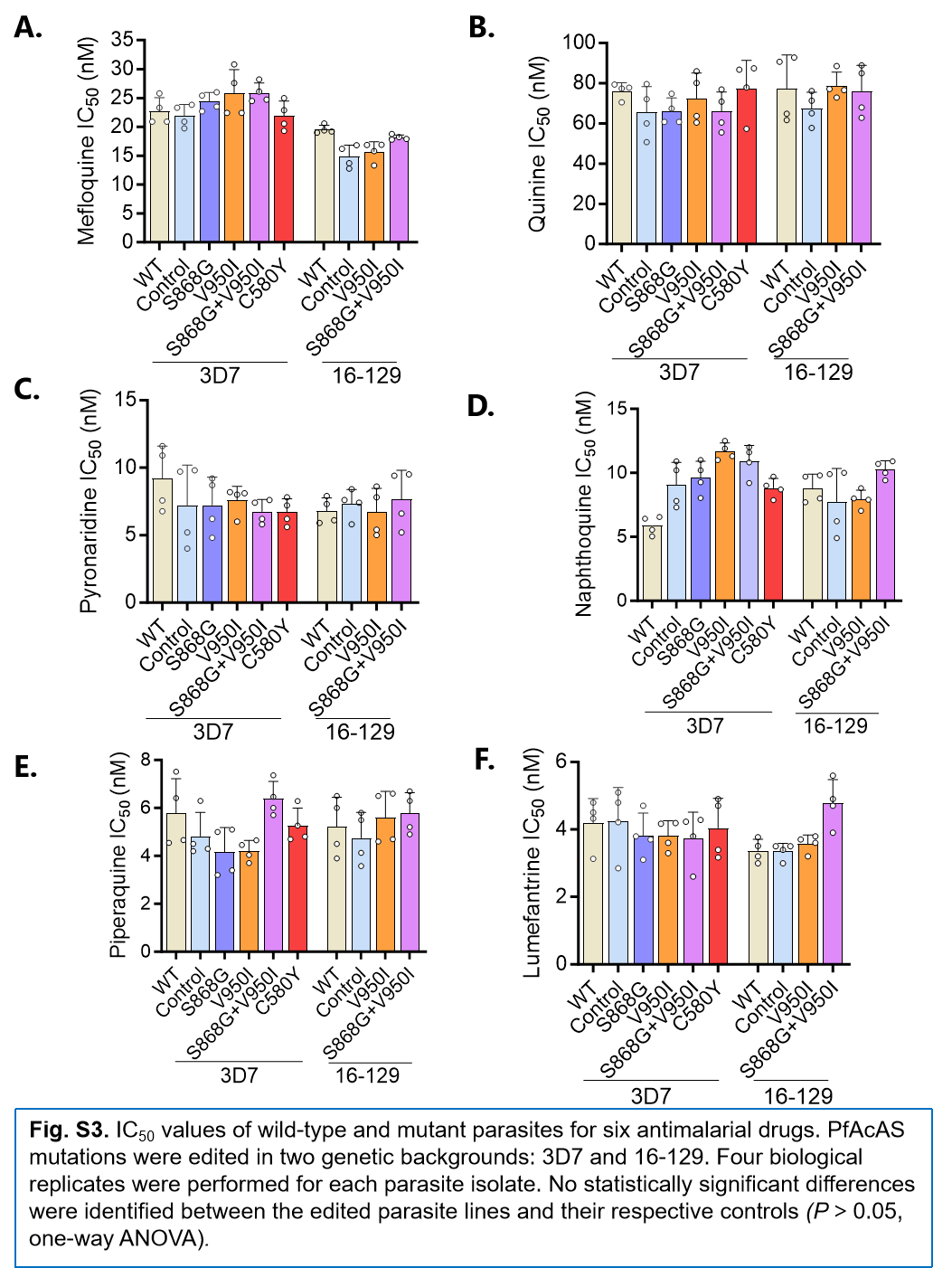

Supplement: Fig. S3 — IC50 values of wild-type and mutant parasites for six antimalarial drugs. [file spectrum.01026-25-s0003.tif]
