# Supplementary material for: Acetyl-CoA synthetase mutations affect the susceptibility of Plasmodium falciparum to antimalarial drugs
Source: Microbiol Spectr. 2025 Sep 11;13(10):e01026-25. doi: 10.1128/spectrum.01026-25 (PMC12502573; doi:10.1128/spectrum.01026-25)
Supplement: Supplemental material — Supplemental figure legends. [file spectrum.01026-25-s0004.docx]

Fig. S1. Manhattan plots showing the significance of SNP association in the GWAS.Values of –log(P) for GWAS were plotted against chromosomal positions of SNPs. Each point represents 1 of 65460 SNPs with MAF >0.01 in a set of 134 isolates. The dashed horizontal line indicates the significance threshold of a P value of p ≤ 7.63×10-7 after Bonferroni correction.

Fig. S2. Homology modeling of PfAcAS reveals that mutations identified are not clustered around the active site of the enzyme or the CoA binding site. Note that the protein structure model has only 940 amino acids. Thus, the position V950I is not labeled in the diagram.

Fig. S3. IC50 values of wild-type and mutant parasites for six antimalarial drugs. PfAcAS mutations were edited in two genetic backgrounds: 3D7 and 16-129. Four biological replicates were performed for each parasite isolate. No statistically significant differences were identified between the edited parasite lines and their respective controls (P > 0.05, one-way ANOVA).
