# Supplementary material for: Acetyl-CoA synthetase mutations affect the susceptibility of Plasmodium falciparum to antimalarial drugs
Source: Microbiol Spectr. 2025 Sep 11;13(10):e01026-25. doi: 10.1128/spectrum.01026-25 (PMC12502573; doi:10.1128/spectrum.01026-25)
Supplement: Supplemental tables — Tables S1 to S7. [file spectrum.01026-25-s0005.docx]

**Supplementary Table 1.** Sample information of 11 recrudescent parasites and 13 ACPR parasites.

|  |  | Sample code | Patients from Region | Diagnosis date  (month-year) | Recrudescent Diagnosis date  (month-year) | Days between episodes |
| --- | --- | --- | --- | --- | --- | --- |
| Recrudescent samples (case group) | | 08-35 | Congo | May-16^#1^ | Jun-16^#2^ | 22 |
| (N=11) |  | 08-16 | Cameroon | Mar-16^#1^ | Apr-16^#1^ | 15 |
|  |  | 16-132 | Ghana | Apr-17^#2^ | May-17^#2^ | 18 |
|  |  | 16-58 | Congo | Jan-17^#2^ | Feb-16^#2^ | 12 |
|  |  | 17-106 | Congo | Aug-17^#2^ | Aug-17^#2^ | 13 |
|  |  | 17-163 | Ghana | Dec-17^#2^ | Dec-17^#2^ | 22 |
|  |  | 17-18 | Ghana | May-17^#2^ | Jun-17^#2^ | 39 |
|  |  | 17-62 | Ghana | Jun-17^#2^ | Jun-17^#2^ | 20 |
|  |  | 18-2 | Ghana | Dec-17^#2^ | Ian-18^#2^ | 37 |
|  |  | 18-34 | Ghana | Feb-18^#2^ | Mar-18^#2^ | 24 |
|  |  | H24 | Ghana | Jan-16^#1^ | Feb-16^#1^ | 21 |
| Samples with adequate clinical and parasitological response (ACPR) (N=13) |  | 16-14 | Ghana | Mar-16^#1^ | - | - |
|  |  | 16-141 | Cameroon | Apr-16^#1^ | - | - |
|  |  | 16-33 | Ghana | Jan-16^#2^ | - | - |
|  |  | 16-34 | Ghana | Jan-16^#2^ | - | - |
|  |  | 16-62 | Ghana | Feb-16^#2^ | - | - |
|  |  | 16-2 | Ghana | Jan-16^#2^ | - | - |
|  |  | 17-109 | Congo | May-17^#2^ | - | - |
|  |  | 17-121 | Cameroon | Jun-17^#2^ | - | - |
|  |  | 17-139 | Ghana | Aug-17^#2^ | - | - |
|  |  | 17-17 | Ghana | Jan-17^#2^ | - | - |
|  |  | 17-19 | Cameroon | Jan-17^#2^ | - | - |
|  |  | 17-29 | Cameroon | Feb-17^#2^ | - | - |
|  |  | 17-83 | Ghana | Feb-17^#2^ | - | - |

Malaria patients were hospitalized in Shanglin County and treated following the guidelines of the Chinese Center for Disease Control and Prevention (CDC) and WHO recommendations on the diagnosis and treatment of uncomplicated and severe malaria. For those *P. falciparum* patients, All were treated with Protocol #1 when first diagnosed: intravenous (IV) injections of Artesunate, Guilin Pharmaceutical (Shanghai) Co. LTD (Approval date: 02/12/2015) for 3 days, then taken ACT(DHA-PPQ) for 3 days.

Day0:120mg (or 2.4mg/kg);12h:120mg (or 2.4mg/kg);

Day1:120mg (or 2.4mg/kg);

Day2:120mg (or 2.4mg/kg).

Maximum Dosage:480mg.

After 3 days of intravenous (IV) injections of Artesunate, ACT(DHA 40mg-PPQ 320mg) was followed: A total of 8 tablets. Take 4 tablets on the first day, and 2 tablets each on the second and third days.

The recrudescent cases were treated with intravenous (IV) injections of Artesunate for 7 days, then taken ACT(DHA-PPQ) for 3 days.

Protocol #2: Artesunate, NHFPCC (Recommendation date: 5/20/2016).

Day0:120mg (or 2.4mg/kg);12h:120mg (or 2.4mg/kg);

Day1:120mg (or 2.4mg/kg);

Day2:120mg (or 2.4mg/kg);

Day3:120mg (or 2.4mg/kg);

Day4:120mg (or 2.4mg/kg);

Day5:120mg (or 2.4mg/kg);

Day6:120mg (or 2.4mg/kg).

Maximum Dosage:960mg.

After 7 days of intravenous (IV) injections of Artesunate, ACT(DHA 40mg-PPQ 320mg) was followed: A total of 8 tablets. Take 4 tablets on the first day, and 2 tablets each on the second and third days.

**Supplementary Table 2.** Sample information of 110 ART-sensitive isolates downloaded from SRA database as the control group.

| SRA id | Region | year | sample_id | ART-susceptivity |
| --- | --- | --- | --- | --- |
| ERR636083 | Ghana | 2014 | QG0436-C | sensitive |
| ERR636091 | Ghana | 2014 | PF1101-C | sensitive |
| ERR636098 | Ghana | 2014 | PF1102-C | sensitive |
| ERR636260 | Ghana | 2014 | PF1103-C | sensitive |
| ERR636261 | Ghana | 2014 | PF1104-C | sensitive |
| ERR636262 | Ghana | 2014 | PF1105-C | sensitive |
| ERR636263 | Ghana | 2014 | PF1107-C | sensitive |
| ERR636264 | Ghana | 2014 | PF1108-C | sensitive |
| ERR636265 | Ghana | 2014 | PF1110-C | sensitive |
| ERR636266 | Ghana | 2014 | PF1111-C | sensitive |
| ERR636267 | Ghana | 2014 | PF1112-C | sensitive |
| ERR636268 | Ghana | 2014 | PF1113-C | sensitive |
| ERR636269 | Ghana | 2014 | PF1114-C | sensitive |
| ERR1214127 | Ghana | 2015 | PF1118-Cx | sensitive |
| ERR1214175 | Ghana | 2015 | PF1119-Cx8 | sensitive |
| ERR1214200 | Ghana | 2015 | PF1120-Cx6 | sensitive |
| ERR1214129 | Ghana | 2015 | PF1123-Cx | sensitive |
| ERR1214130 | Ghana | 2015 | PF1124-Cx | sensitive |
| ERR1214131 | Ghana | 2015 | PF1125-Cx | sensitive |
| ERR1214132 | Ghana | 2015 | PF1126-Cx | sensitive |
| ERR1214201 | Ghana | 2015 | PF1127-Cx7 | sensitive |
| ERR1214133 | Ghana | 2015 | PF1128-Cx | sensitive |
| ERR1214177 | Ghana | 2015 | PF1129-Cx9 | sensitive |
| ERR1214178 | Ghana | 2015 | PF1131-Cx9 | sensitive |
| ERR1214135 | Ghana | 2015 | PF1132-Cx | sensitive |
| ERR1214136 | Ghana | 2015 | PF1134-Cx | sensitive |
| ERR1214137 | Ghana | 2015 | PF1135-Cx | sensitive |
| ERR1214138 | Ghana | 2015 | PF1136-Cx | sensitive |
| ERR1214139 | Ghana | 2015 | PF1137-Cx | sensitive |
| ERR1214140 | Ghana | 2015 | PF1139-Cx | sensitive |
| ERR1214141 | Ghana | 2015 | PF1140-Cx | sensitive |
| ERR1214142 | Ghana | 2015 | PF1141-Cx | sensitive |
| ERR1214143 | Ghana | 2015 | PF1142-Cx | sensitive |
| ERR1214183 | Ghana | 2015 | PF1148-Cx8 | sensitive |
| ERR1214184 | Ghana | 2015 | PF1150-Cx9 | sensitive |
| ERR1214206 | Ghana | 2015 | PF1151-Cx6 | sensitive |
| ERR1214186 | Ghana | 2015 | PF1152-Cx9 | sensitive |
| ERR1214187 | Ghana | 2015 | PF1153-Cx9 | sensitive |
| ERR1214147 | Ghana | 2015 | PF1154-Cx | sensitive |
| ERR1214148 | Ghana | 2015 | PF1155-Cx | sensitive |
| ERR1214189 | Ghana | 2015 | PF1157-Cx9 | sensitive |
| ERR1214190 | Ghana | 2015 | PF1160-Cx9 | sensitive |
| ERR1214191 | Ghana | 2015 | PF1161-Cx8 | sensitive |
| ERR1214149 | Ghana | 2015 | PF1165-Cx | sensitive |
| ERR1214150 | Ghana | 2015 | PF1167-Cx | sensitive |
| ERR1214151 | Ghana | 2015 | PF1169-Cx | sensitive |
| ERR1214152 | Ghana | 2015 | PF1170-Cx | sensitive |
| ERR1214153 | Ghana | 2015 | PF1171-Cx | sensitive |
| ERR1214154 | Ghana | 2015 | PF1172-Cx | sensitive |
| ERR1214155 | Ghana | 2015 | PF1173-Cx | sensitive |
| ERR1214156 | Ghana | 2015 | PF1174-Cx | sensitive |
| ERR1214157 | Ghana | 2015 | PF1175-Cx | sensitive |
| ERR1214158 | Ghana | 2015 | PF1176-Cx | sensitive |
| ERR1214159 | Ghana | 2015 | PF1177-Cx | sensitive |
| ERR1214160 | Ghana | 2015 | PF1178-Cx | sensitive |
| ERR1214195 | Ghana | 2015 | PF1179-Cx8 | sensitive |
| ERR1214161 | Ghana | 2015 | PF1180-Cx | sensitive |
| ERR1214162 | Ghana | 2015 | PF1182-Cx | sensitive |
| ERR1214163 | Ghana | 2015 | PF1184-Cx | sensitive |
| ERR1214164 | Ghana | 2015 | PF1185-Cx | sensitive |
| ERR1214165 | Ghana | 2015 | PF1187-Cx | sensitive |
| ERR1214166 | Ghana | 2015 | PF1188-Cx | sensitive |
| ERR1214167 | Ghana | 2015 | PF1189-Cx | sensitive |
| ERR1214168 | Ghana | 2015 | PF1190-Cx | sensitive |
| ERR1214169 | Ghana | 2015 | PF1194-Cx | sensitive |
| ERR1214170 | Ghana | 2015 | PF1195-Cx | sensitive |
| ERR1214171 | Ghana | 2015 | PF1196-Cx | sensitive |
| ERR1214197 | Ghana | 2015 | PF1197-Cx8 | sensitive |
| ERR1214198 | Ghana | 2015 | PF1198-Cx8 | sensitive |
| ERR1214174 | Ghana | 2015 | PF1200-Cx | sensitive |
| ERR1514577 | Congo | 2014 | QG0383-C | sensitive |
| ERR1514578 | Congo | 2014 | QG0386-C | sensitive |
| ERR1514579 | Congo | 2014 | QG0396-C | sensitive |
| ERR1514580 | Congo | 2014 | QG0397-C | sensitive |
| ERR1514581 | Congo | 2014 | QG0398-C | sensitive |
| ERR1514582 | Congo | 2014 | QG0399-C | sensitive |
| ERR1514583 | Congo | 2014 | QG0400-C | sensitive |
| ERR1514588 | Congo | 2014 | QG0411-C | sensitive |
| ERR1514589 | Congo | 2014 | QG0412-C | sensitive |
| ERR1514590 | Congo | 2014 | QG0413-C | sensitive |
| ERR1514591 | Congo | 2014 | QG0414-C | sensitive |
| ERR1514594 | Congo | 2014 | QG0419-C | sensitive |
| ERR1514596 | Congo | 2014 | QG0422-C | sensitive |
| ERR1514597 | Congo | 2014 | QG0423-C | sensitive |
| ERR1514598 | Congo | 2014 | QG0424-C | sensitive |
| ERR1514599 | Congo | 2014 | QG0426-C | sensitive |
| ERR1514600 | Congo | 2014 | QG0430-C | sensitive |
| ERR1514601 | Congo | 2014 | QG0431-C | sensitive |
| ERR1514602 | Congo | 2014 | QG0435-C | sensitive |
| ERR1514603 | Congo | 2014 | QG0436-C | sensitive |
| ERR1514604 | Congo | 2014 | QG0438-C | sensitive |
| ERR1514605 | Congo | 2014 | QG0439-C | sensitive |
| ERR1514607 | Congo | 2014 | QG0442-C | sensitive |
| ERR1514608 | Congo | 2014 | QG0443-C | sensitive |
| ERR1514609 | Congo | 2014 | QG0444-C | sensitive |
| ERR1514610 | Congo | 2014 | QG0445-C | sensitive |
| ERR1514612 | Congo | 2014 | QG0447-C | sensitive |
| ERR1514613 | Congo | 2014 | QG0448-C | sensitive |
| ERR1514614 | Congo | 2014 | QG0449-C | sensitive |
| ERR1514615 | Congo | 2014 | QG0450-C | sensitive |
| ERR580582 | Cameroon | 2013 | QP0233-C | sensitive |
| ERR580472 | Cameroon | 2013 | QP0234-C | sensitive |
| ERR562829 | Cameroon | 2013 | QP0235-C | sensitive |
| ERR580473 | Cameroon | 2013 | QP0236-C | sensitive |
| ERR580509 | Cameroon | 2013 | QP0237-C | sensitive |
| ERR580542 | Cameroon | 2013 | QP0238-C | sensitive |
| ERR580580 | Cameroon | 2013 | QP0239-C | sensitive |
| ERR580544 | Cameroon | 2013 | QP0240-C | sensitive |
| ERR580545 | Cameroon | 2013 | QP0241-C | sensitive |
| ERR580471 | Cameroon | 2013 | QP0242-C | sensitive |

**Supplementary Table 3.** Frequencies of non-synonymous mutations frequency in *P. falciparum* in the 11 recrudescent samples (case group) , 123 control samples and public database.

| **Chromosome and  mutation position** | **Cases group** | **Control group** | ***P*** value （<7.63×10^-7^） | **public database** |
| --- | --- | --- | --- | --- |
| Pf3D7_01_v3:343274 | 0.3333 | 0.025 | 9.315×10-8 | 0.04 |
| Pf3D7_01_v3:354708 | 0.3333 | 0 | 1.39×10-19 | 0 |
| Pf3D7_01_v3:509112 | 0.3333 | 0.025 | 9.315×10-8 | 0.05 |
| Pf3D7_01_v3:547792 | 0.4 | 0.03478 | 2.131×10-7 | 0.1 |
| Pf3D7_01_v3:547793 | 0.4 | 0.03478 | 2.131×10-7 | 0.1 |
| Pf3D7_01_v3:561056 | 0.3333 | 0.01639 | 7.254×10-10 | 0 |
| Pf3D7_02_v3:196302 | 0.3333 | 0.02479 | 8.116×10-8 | 0.02 |
| Pf3D7_02_v3:507659 | 0.2857 | 0.01639 | 1.571×10-8 | 0.02 |
| Pf3D7_02_v3:663867 | 0.25 | 0 | 4.535×10-15 | 0 |
| Pf3D7_03_v3:149247 | 0.3333 | 0.008197 | 3.644×10-13 | 0.01 |
| Pf3D7_03_v3:400253 | 0.4286 | 0.04959 | 6.949×10-8 | 0.05 |
| Pf3D7_03_v3:456899 | 0.375 | 0.04132 | 8.338×10-8 | 0.23 |
| Pf3D7_03_v3:592187 | 0.2857 | 0.008264 | 2.53×10-11 | 0.01 |
| Pf3D7_03_v3:796549 | 0.3333 | 0.02459 | 7.072×10-8 | 0.01 |
| Pf3D7_03_v3:924115 | 0.6667 | 0.1066 | 2.383×10-8 | 0.26 |
| Pf3D7_04_v3:270767 | 0.5 | 0.03306 | 4.566×10-12 | 0.05 |
| Pf3D7_04_v3:317532 | 0.2857 | 0.008264 | 2.53×10-11 | 0.01 |
| Pf3D7_04_v3:408806 | 0.3333 | 0.01653 | 8.608×10-10 | 0.02 |
| Pf3D7_04_v3:850273 | 0.3333 | 0.01639 | 7.254×10-10 | 0.01 |
| Pf3D7_04_v3:1100511 | 0.2857 | 0.01639 | 1.571×10-8 | 0.04 |
| Pf3D7_04_v3:1130328 | 0.5 | 0.05785 | 2.843×10-8 | 0.05 |
| Pf3D7_05_v3:78122 | 0.6 | 0.02439 | 3.13×10-14 | 0.18 |
| Pf3D7_05_v3:300612 | 0.2857 | 0 | 3.929×10-17 | 0 |
| Pf3D7_05_v3:323671 | 0.2857 | 0.008264 | 2.53×10-11 | 0.02 |
| Pf3D7_05_v3:852072 | 0.2857 | 0.01653 | 1.821×10-8 | 0.03 |
| Pf3D7_05_v3:1042212 | 0.2857 | 0.01653 | 1.821×10-8 | 0.01 |
| Pf3D7_05_v3:1141051 | 0.2857 | 0.008197 | 2.082×10-11 | 0.01 |
| Pf3D7_05_v3:1285662 | 0.2857 | 0 | 5.25×10-17 | 0 |
| Pf3D7_06_v3:103244 | 0.3333 | 0.008264 | 4.57×10-13 | 0.06 |
| Pf3D7_06_v3:125883 | 0.8 | 0.1468 | 1.093×10-7 | 0.09 |
| Pf3D7_06_v3:869449 | 0.4286 | 0.05738 | 4.398×10-7 | 0.05 |
| Pf3D7_06_v3:1108821 | 0.25 | 0.008264 | 5.179×10-10 | 0.01 |
| **Pf3D7_06_v3:1114690(S868G)** | **0.2857** | **0.008264** | 2.53×10-11 | **0.01** |
| **Pf3D7_06_v3:1114936(V950I)** | **0.25** | **0.008264** | 5.179×10-10 | **0.01** |
| Pf3D7_07_v3:90501 | 0.2857 | 0.01639 | 1.571×10-8 | 0.01 |
| Pf3D7_07_v3:340062 | 0.25 | 0.01653 | 1.81×10-7 | 0.25 |
| Pf3D7_07_v3:340077 | 0.25 | 0.01653 | 1.81×10-7 | 0.01 |
| Pf3D7_07_v3:340092 | 0.25 | 0.008403 | 7.291×10-10 | 0.01 |
| Pf3D7_07_v3:483522 | 0.25 | 0.008197 | 4.365×10-10 | 0 |
| Pf3D7_07_v3:609499 | 0.375 | 0 | 3.647×10-21 | 0.12 |
| Pf3D7_07_v3:609748 | 0.2857 | 0 | 3.929×10-17 | 0.01 |
| Pf3D7_07_v3:710518 | 0.2857 | 0.01639 | 1.571×10-8 | 0.01 |
| Pf3D7_07_v3:897916 | 0.4286 | 0.04918 | 5.995×10-8 | 0.3 |
| Pf3D7_07_v3:918600 | 0.2857 | 0.01639 | 1.571×10-8 | 0.01 |
| Pf3D7_08_v3:255907 | 0.3333 | 0.02479 | 8.116×10-8 | 0.02 |
| Pf3D7_08_v3:257600 | 0.2857 | 0.01653 | 1.821×10-8 | 0.04 |
| Pf3D7_08_v3:264553 | 0.25 | 0.01653 | 1.81×10-7 | 0.03 |
| Pf3D7_08_v3:407408 | 0.2857 | 0.01653 | 1.821×10-8 | 0.01 |
| Pf3D7_08_v3:920399 | 0.2222 | 0.008264 | 5.455×10-9 | 0.05 |
| Pf3D7_08_v3:1115173 | 0.25 | 0 | 4.535×10-15 | 0 |
| Pf3D7_08_v3:1133076 | 0.2857 | 0 | 5.25×10-17 | 0.07 |
| Pf3D7_08_v3:1173499 | 0.5714 | 0.1074 | 6.567×10-7 | 0.14 |
| Pf3D7_08_v3:1194559 | 0.4 | 0.02542 | 4.228×10-9 | 0.32 |
| Pf3D7_08_v3:1310920 | 0.2857 | 0.008197 | 2.082×10-11 | 0.02 |
| Pf3D7_08_v3:1327621 | 0.5 | 0.04202 | 2.539×10-10 | 0.03 |
| Pf3D7_08_v3:1327624 | 0.5 | 0.04167 | 2.095×10-10 | 0.03 |
| Pf3D7_09_v3:314119 | 0.3333 | 0 | 1.391×10-19 | 0 |
| Pf3D7_09_v3:340812 | 0.2857 | 0.008264 | 2.53×10-11 | 0.03 |
| Pf3D7_09_v3:505952 | 0.25 | 0 | 4.535×10-15 | 0 |
| Pf3D7_09_v3:836819 | 0.2857 | 0 | 3.929×10-17 | 0 |
| Pf3D7_09_v3:932451 | 0.3333 | 0.02459 | 7.072×10-8 | 0.05 |
| Pf3D7_09_v3:1095983 | 0.2857 | 0.01653 | 1.821×10-8 | 0.04 |
| Pf3D7_09_v3:1281876 | 0.2857 | 0 | 5.25×10-17 | 0 |
| Pf3D7_10_v3:529282 | 0.2857 | 0 | 5.25×10-17 | 0.01 |
| Pf3D7_10_v3:1166708 | 0.25 | 0.01639 | 1.59×10-7 | 0.04 |
| Pf3D7_10_v3:1404483 | 0.3333 | 0.02752 | 4.251×10-7 | 0 |
| Pf3D7_10_v3:1404486 | 0.3333 | 0.02778 | 4.881×10-7 | 0 |
| Pf3D7_11_v3:115167 | 0.3333 | 0.01653 | 8.608×10-10 | 0 |
| Pf3D7_11_v3:191621 | 0.3333 | 0.01667 | 1.021×10-9 | 0.01 |
| Pf3D7_11_v3:254794 | 0.5 | 0.03306 | 4.566×10-12 | 0.01 |
| Pf3D7_11_v3:690214 | 0.3333 | 0.01639 | 7.254×10-10 | 0.06 |
| Pf3D7_11_v3:1369739 | 0.4286 | 0.01653 | 1.019×10-14 | 0.01 |
| Pf3D7_11_v3:1626383 | 0.2857 | 0.01653 | 1.821×10-8 | 0.06 |
| Pf3D7_11_v3:1879210 | 0.2857 | 0 | 5.25×10-17 | 0 |
| Pf3D7_12_v3:233225 | 0.2857 | 0.008197 | 2.082×10-11 | 0.01 |
| Pf3D7_12_v3:233413 | 0.2857 | 0.008264 | 2.53×10-11 | 0.02 |
| Pf3D7_12_v3:659931 | 0.25 | 0.01653 | 1.81×10-7 | 0.01 |
| Pf3D7_12_v3:715524 | 0.3333 | 0.02479 | 8.116×10-8 | 0.09 |
| Pf3D7_12_v3:1176149 | 0.3333 | 0.01639 | 7.254×10-10 | 0 |
| Pf3D7_12_v3:1449221 | 0.2857 | 0.01639 | 1.571×10-8 | 0.03 |
| Pf3D7_12_v3:1976328 | 0.3333 | 0.01639 | 7.254×10-10 | 0.01 |
| Pf3D7_12_v3:2067440 | 0.3333 | 0.01653 | 8.608×10-10 | 0.04 |
| Pf3D7_13_v3:474161 | 0.4 | 0.03361 | 1.227×10-7 | 0.04 |
| Pf3D7_13_v3:536730 | 0.5714 | 0.05738 | 2.121×10-11 | 0.06 |
| Pf3D7_13_v3:559768 | 0.25 | 0.008264 | 5.179×10-10 | 0.03 |
| Pf3D7_13_v3:615253 | 0.2857 | 0.008197 | 2.082×10-11 | 0.01 |
| Pf3D7_13_v3:1012257 | 0.3333 | 0.008403 | 7.19×10-13 | 0.01 |
| Pf3D7_13_v3:1175241 | 0.2857 | 0.008264 | 2.53×10-11 | 0.02 |
| Pf3D7_13_v3:1311810 | 0.2857 | 0.01639 | 1.571×10-8 | 0 |
| Pf3D7_13_v3:2085113 | 0.2857 | 0.01639 | 1.571×10-8 | 0.01 |
| Pf3D7_13_v3:2277466 | 0.2857 | 0.008264 | 2.53×10-11 | 0.02 |
| Pf3D7_14_v3:329949 | 0.5714 | 0.02479 | 2.207×10-18 | 0.03 |
| Pf3D7_14_v3:341836 | 0.25 | 0 | 3.517×10-15 | 0 |
| Pf3D7_14_v3:646419 | 0.3333 | 0 | 1.949×10-19 | 0 |
| Pf3D7_14_v3:768719 | 0.4 | 0.03279 | 8.116×10-8 | 0.02 |
| Pf3D7_14_v3:1355727 | 0.2857 | 0.01653 | 1.821×10-8 | 0.04 |
| Pf3D7_14_v3:1900413 | 0.4286 | 0.02479 | 3.668×10-12 | 0.03 |
| Pf3D7_14_v3:2223179 | 0.2857 | 0.008264 | 2.53×10-11 | 0.01 |
| Pf3D7_14_v3:3000246 | 0.2857 | 0.008264 | 2.53×10-11 | 0.01 |
| Pf3D7_14_v3:3097632 | 0.4 | 0.025 | 3.043×10-9 | 0.05 |
| Pf3D7_14_v3:3235252 | 0.3333 | 0.01639 | 7.254×10-10 | 0.01 |

**Supplementary Table 4.** GO enrichment analysis of genes with known functions (biological process, cellular component and molecular function).

| **Gene Ontology** | **ID** | **Name** | **Result gene list** |
| --- | --- | --- | --- |
| Biological Process | GO:0051169 | nuclear transport | PF3D7_0627700, PF3D7_0716100, PF3D7_1473700, |
|  | GO:0006325 | chromatin organization | PF3D7_0216000, PF3D7_0627800, PF3D7_1228800, |
|  | GO:0006357 | regulation of transcription by RNA polymerase II | PF3D7_0216000, PF3D7_1140700, PF3D7_1473700, |
|  | GO:0045185 | maintenance of protein location | PF3D7_0321900, PF3D7_1013500, |
|  | GO:0034504 | protein localization to nucleus | PF3D7_0627700, PF3D7_1473700, |
|  | GO:0034030 | ribonucleoside bisphosphate biosynthetic process | PF3D7_0627800, PF3D7_1102400, |
|  | GO:0032065 | maintenance of protein location in cell cortex | PF3D7_1013500 |
|  | GO:1990126 | retrograde transport, endosome to plasma membrane | PF3D7_0720700 |
|  | GO:0019427 | acetyl-CoA biosynthetic process from acetate | PF3D7_0627800, |
|  | GO:0043137 | DNA replication, removal of RNA primer | PF3D7_0408500, |
|  | GO:0090158 | endoplasmic reticulum membrane organization | PF3D7_1416100, |
|  | GO:0034613 | cellular protein localization | PF3D7_0707300, |
|  | GO:0032988 | ribonucleoprotein complex disassembly | PF3D7_0531400, |
|  | GO:0018095 | protein polyglutamylation | PF3D7_1147200, |
|  | GO:0045732 | positive regulation of protein catabolic process | PF3D7_0826100, |
|  | GO:0072595 | maintenance of protein localization in organelle | PF3D7_0321900, |
|  | GO:0030522 | intracellular receptor signaling pathway | PF3D7_1475400, |
|  | GO:0007004 | telomere maintenance via telomerase | PF3D7_1314200, |
|  |  |  |  |
|  |  |  |  |
|  |  |  |  |
|  |  |  |  |
| Cellular Component | GO:0012505 | endomembrane system | PF3D7_0321900,PF3D7_0525100,PF3D7_0627700,PF3D7_0707300,PF3D7_0710800,PF3D7_0720700,PF3D7_1416100,PF3D7_1473700, |
|  | GO:0045177 | apical part of cell | PF3D7_0405900,PF3D7_0424400,PF3D7_0707300,PF3D7_1216600,PF3D7_1312800, |
|  | GO:0009986 | cell surface | PF3D7_0424400,PF3D7_0830800,PF3D7_0831100,PF3D7_1035400, PF3D7_1475400, |
|  | GO:0044428 | obsolete nuclear part | PF3D7_0310500,PF3D7_0408500,PF3D7_0716100,PF3D7_1314200, |
|  | GO:0020007 | apical complex | PF3D7_0405900,PF3D7_0424400,PF3D7_0707300,PF3D7_1216600, |
|  | GO:0031225 | anchored component of membrane | PF3D7_0405900,PF3D7_0707300,PF3D7_1475400, |
|  | GO:0044228 | host cell surface | PF3D7_0424400,PF3D7_0830800,PF3D7_0831100, |
|  | GO:0031226 | intrinsic component of plasma membrane | PF3D7_0319000,PF3D7_0405900,PF3D7_1475400, |
|  | GO:0020008 | rhoptry | PF3D7_0405900,PF3D7_0424400,PF3D7_0707300, |
|  | GO:0005643 | nuclear pore | PF3D7_0627700,PF3D7_1473700, |
|  | GO:1990225 | rhoptry neck | PF3D7_0405900,PF3D7_0424400, |
|  | GO:0005615 | extracellular space | PF3D7_0501400,PF3D7_0507200, |
|  | GO:0046658 | anchored component of plasma membrane | PF3D7_0405900,PF3D7_1475400, |
|  | GO:0000333 | telomerase catalytic core complex | PF3D7_1314200, |
|  | GO:0033186 | CAF-1 complex | PF3D7_1228800, |
|  | GO:0071008 | U2-type post-mRNA release spliceosomal complex | PF3D7_0531400, |
|  | GO:0044613 | nuclear pore central transport channel | PF3D7_1473700, |
|  | GO:0005769 | early endosome | PF3D7_0720700, |
|  | GO:0098857 | membrane microdomain | PF3D7_0707300, |
|  | GO:0031233 | external side of plasma membrane | PF3D7_1475400, |
|  | GO:0016514 | SWI/SNF complex | PF3D7_0216000, |
| Molecular Function | GO:0061630 | ubiquitin protein ligase activity | PF3D7_0314700,PF3D7_0826100,PF3D7_1475400, |
|  | GO:0003924 | GTPase activity | PF3D7_0602400,PF3D7_0827100,PF3D7_1416100, |
|  | GO:0016881 | acid-amino acid ligase activity | PF3D7_1102400,PF3D7_1147200, |
|  | GO:0016405 | CoA-ligase activity | PF3D7_0525100,PF3D7_0627800, |
|  | GO:0005543 | phospholipid binding | PF3D7_0720700,PF3D7_1013500, |
|  | GO:0000287 | magnesium ion binding | PF3D7_0319000,PF3D7_0408500, |
|  | GO:0140345 | phosphatidylcholine flippase activity | PF3D7_0319000, |
|  | GO:0003720 | telomerase activity | PF3D7_1314200, |
|  | GO:0005488 | binding | PF3D7_0405900, |
|  | GO:0004629 | phospholipase C activity | PF3D7_1013500, |
|  | GO:0017056 | structural constituent of nuclear pore | PF3D7_1473700, |
|  | GO:0004867 | serine-type endopeptidase inhibitor activity | PF3D7_0501400, |
|  | GO:0004835 | tubulin-tyrosine ligase activity | PF3D7_1147200, |
|  | GO:0004632 | phosphopantothenate--cysteine ligase activity | PF3D7_1102400, |
|  | GO:0003987 | acetate-CoA ligase activity | PF3D7_0627800, |
|  | GO:0062072 | H3K9me3 modified histone binding | PF3D7_1140700, |
|  | GO:0030507 | spectrin binding | PF3D7_0424400, |
|  | GO:0061608 | nuclear import signal receptor activity | PF3D7_0627700, |

**Supplementary Table 5.** *In vitro* susceptibilities (IC_50_ in nM) and RSA of engineered strains in 3D7 and 16-129 background strains.

| Drugs/RSA | Parasite Strains (Mean±SD) | | | | | | | | | | |
| --- | --- | --- | --- | --- | --- | --- | --- | --- | --- | --- | --- |
|  | 3D7-WT | 3D7control | 3D7S868G | 3D7V950I | 3D7S868G+V950I | 3D7C580Y | 16-129-WT | 16-129control | 16-129V950I | 16-129S868G+V950I | Cutoff values |
| DHA | 0.64±0.11 | 0.76±0.12 | 1.76±0.30^d^ | 1.01±0.04 | 1.75±0.22^d^ | 0.79±0.16 | 1.13±0.38 | 1.05±0.25 | 1.49±0.29 | 1.92±0.25^d^ | - |
| AS | 1.52±0.34 | 1.11±0.18 | 4.48±0.98^d^ | 3.88±0.96^d^ | 3.78±0.62^c^ | 5.05±0.83^d^ | 4.03±0.33 | 3.08±0.71 | 3.32±0.86 | 4.08±0.26 | - |
| AM | 2.47±0.96 | 1.14±0.37 | 3.29±1.18^a^ | 2.54±0.80 | 3.12±0.92 | 3.23±0.26^a^ | 2.70±1.33 | 2.27±0.59 | 1.79±0.29 | 1.97±1.08 | - |
| CQ | 14.78±1.42 | 14.66±1.62 | 22.50±5.05^a^ | 17.65±0.90 | 22.67±4.10^a^ | 20.17±5.86 | 14.80±2.05 | 11.93±3.16 | 13.5±1.81 | 14.44±2.27 | 100 |
| MFQ | 22.73±2.34 | 22.94±1.93 | 24.40±1.56 | 25.80±4.14 | 25.87±1.71 | 21.95±2.52 | 19.55±0.72 | 14.88±1.93 | 15.72±1.68 | 18.17±0.43 | 30 |
| QN | 76.28±3.95 | 65.59±12.81 | 65.93±6.69 | 72.59±12.44 | 65.94±9.72 | 77.34±14.05 | 77.50±16.53 | 67.55±7.96 | 78.79±6.75 | 76.15±12.70 | 600 |
| PND | 9.21±2.39 | 7.18±3.01 | 7.23±2.08 | 7.58±1.05 | 6.75±0.89 | 6.70±0.99 | 6.80±0.96 | 7.30±1.09 | 6.70±1.77 | 7.68±2.13 | 15 |
| NQ | 5.89±0.72 | 9.05±1.77 | 9.63±1.27 | 11.68±0.67 | 10.91±1.24 | 8.82±0.76 | 8.80±1.11 | 7.76±2.60 | 7.94±0.72 | 10.26±0.68 | - |
| PPQ | 5.78±1.43 | 4.83±0.99 | 4.18±1.01 | 4.23±0.43 | 6.38±0.72 | 5.26±0.73 | 5.23±0.73 | 4.75±1.07 | 5.60±1.10^a^ | 5.78±0.86 | - |
| LMF | 4.19±0.73 | 4.24±0.99 | 3.83±0.66 | 3.82±0.44 | 3.73±0.78 | 4.04±0.88 | 3.37±0.34 | 3.35±0.24 | 3.58±0.26 | 4.78±0.70 | - |
| RSA(%) | 0.44±0.11 | 0.24±0.14 | 0.29±0.10 | 0.34±0.05 | 0.59±0.05 | 2.68±+0.59^c^ | 0.78±0.09 | 0.56±0.06 | 0.50±0.03 | 0.51±0.10 | 1% |
|  |  |  |  |  |  |  |  |  |  |  |  |

Annotation: Dihydroartemisinin (DHA), artesunate (AS), artemether (AM), chloroquine (CQ), mefloquine (MFQ), quinine (QN), pyronaridine (PND), naphthoquine (NQ), piperaquine (PPQ) and lumefantrine (LMF). RSA: RSA values are percentages (%). The ring survival rates (RSA) were determined by comparing surviving parasites in DHA-treated with those in vehicle-treated wells. Data represent the mean±standard deviation of the results. Four biological and technical replicates were performed for each parasite isolate. Statistical comparisons were made using one-way ANOVA test, compared the engineered strains to corresponding control in two genetic backgrounds. “a” represented *P*＜0.05, “c” represented *P*＜0.001. “d” represented *P*＜0.0001“-” represented there are no data were reported.

**Supplementary Table 6.** The gRNA primers of PfAcAS.

| **Primer name** | **Sequences** |
| --- | --- |
| PfAcAS-gRNA-F | 5'-CATATTAAGTATATAATATTGATGGATACTATTGGATATCGTTTTAGAGCTAGAAATAGC-3' |
| PfAcAS-gRNA-R | 5'-GCTATTTCTAGCTCTAAAACGATATCCAATAGTATCCATCAATATTATATACTTAATATG-3' |

The blue shade represents the sequence of gRNA (20 bp); the sequences at both ends represent homologous arms to insert into the plasmid.

**Supplementary Table 7.** The primers carrying PfAcAS S868G or V950I for constructing donors.

| fragment | Primer name | sequences |
| --- | --- | --- |
| Outer | P1CoA-Out-F | 5'-GGTGTTGCACATACAACAGCTGG -3' |
|  | P2 3D7-W-R | 5'-CGCTTGCAGCTGTACAAAAATAATC -3' |
| *PfAcAS*  D1  0  (563bp) | P3 CoA-D1-F1 | 5'-gctgcggccctagtctagggcgcgccGGAGTCTAATACAAACAC -3' |
|  | P5 CoA-D1-R1 | 5'-CCGGAGATCCAGTAATATCCATCTTCATCTCTGAACGC -3' |
| *PfAcAS*  D2  (217bp) | P6 CoA-D2-F2 | 5'-GATGGATATTACTGGATCTCCGGACGAATCGATGATAC -3' |
|  | P7 CoA-D2-R2 | 5'-CTGTAATACCTTTACcATAATTTTTTATGTCTCC -3' |
| *PfAcAS*  D3  (367bp) | P8 CoA-D3-F3 | 5'-CATAAAAAATTATgGTAAAGGTATTACAGATATTG -3' |
|  | P122848-OUT-D2-R 2848-OUT-D2-R2848-OUT-D2-R | 5'- ttaattttttttacaaaatgcttaagGATTGTTAGTTAATTGATTTG -3' |
| *PfAcAS*-V950I | P10 2848-D1-R | 5'-CATCTTAATATTCTTCTAAtAATTTTTCCACTTCTGG-3' |
|  | P11 2848-D2-F | 5'-CCAGAAGTGGAAAAATTaTTAGAAGAATATTAAGATG-3' |

The blue shade represents the sequences of gRNA, and the red shade represents synonymous mutations to gRNA (shielded mutation). The green shade represents that mutation at the 2602 base and 2848 base to achieve a missense mutation of the 868 codon (S868G) and 950 codon (V950I). The yellow shade represents homologous arms to insert into plasmid.
